# Cachiporrini, a remarkable new tribe of Lamprosomatinae (Coleoptera, Chrysomelidae)  from South America

**DOI:** 10.3897/zookeys.78.980

**Published:** 2011-01-29

**Authors:** Maria Lourdes Chamorro, Alexander S. Konstantinov

**Affiliations:** 1 Department of Entomology, National Museum of Natural History, Smithsonian Institution, P.O. Box 37012, MRC-187, Washington, DC, 20013-7012; 2 Systematic Entomology Laboratory, USDA, NMNH, Smithsonian Institution, P.O. Box 37012, MRC-168, Washington, DC, 20013-7012

**Keywords:** New tribe, new genus, new species, antennal clubs, capitulum, leaf beetles, Brazil, phylogeny

## Abstract

A new genus and species of Lamprosomatinae, Cachiporra extremaglobosa Chamorro & Konstantinov, is described from Brazil. A new tribe, Cachiporrini, is proposed. The first phylogenetic analysis of Lamprosomatinae based on adult morphological caharacters is conducted. Comparisons are made among lamprosomatine tribes and genera. A key to tribes is provided.

## Introduction

As taxonomists we can only hope to make the kind of discoveries that prompt suspended awe and prolonged excitement. Such was our reaction when we stumbled upon this new lamprosomatine genus buried within the Cryptocephalinae addenda drawers of the Coleoptera collection at the National Museum of Natural History (NMNH), Smithsonian Institution. Confirmation of this taxon as new was facilitated by the comprehensive chrysomelid collection at NMNH, by the relatively small number of higher-level taxa in Lamprosomatinae, and by the comprehensive studies by (Monrós (1956, 1958) on the group.

Monrós devoted much of his short life and taxonomic expertise to the study of this group of small, round, and shiny beetles. Therefore, it was surprising and somewhat ironic that these specimens are from the Monrós collection acquired by the NMNH in 1959. It is fair to state however, that the Monrós collection is extensive and would require many consecutive lifetimes to study it all.

Lamprosomatinae consists of four tribes and 14 genera (Table 1): Cachiporrini Chamorro & Konstantinov, 2011 (1 genus); Neochlamysini Monrós, 1958 (2 genera); Sphaerocharini Clavareau, 1913 (1 genus); and Lamprosomatini Larcordaire, 1848 (10 genera) (Seeno and Wilcox 1982). Sphaerocharini has been treated as an independent subfamily (Chapuis 1874; Kasap and Crowson 1976), as a tribe of Lamprosomatinae (Lacordaire 1848; Clavareau 1913; Monrós 1956) or as a tribe of the former subfamily Chlamisinae (now a tribe in Cryptocephalinae) (Chen 1940). Monrós (1956) correctly argued for: 1) accepting Sphaerocharini as a tribe of Lamprosomatinae and rejecting the notion that Sphaerocharis represents a transitional group between chlamisines and lamprosomatines and 2) not placing it in its own subfamily. Including Sphaerocharini in Lamprosomatinae is now the prevailing classification (Seeno and Wilcox 1982; Lawrence and Newton 1995). Monrós (1958) later transferred Pseudolychnophaes and Neochlamys from Sphaerocharini to a new tribe, Neochlamysini.

**Table 1.** Summary of classification and distribution of Lamprosomatinae. **NA** Nearctic **NT** Neotropical **AT** Afrotropical **PA** Palearctic **OR** Oriental **AU** Australasian.

Tribe	Genus	Author	Year	NA	NT	AT	PA	OR	AU
Cachiporrini	Cachiporra	Chamorro & Konstantinov	2011		×				
Neochlamysini	Pseudolychnophaes	Achard	1914			×			
	Neochlamys	Jacoby	1882			×			
Sphaerocharini	Sphaerocharis	Lacordaire	1848		×				
Lamprosomatini	Xenoomorphus	Monrós	1956			×			
	Oyarzuna	Bechyné	1950		×				
	Oomorphus	Curtis	1831	×	×	×	×	×	×
	Asisia	Bezdek, Löbl, Konstantinov	2010					×	
	Oomorphoides	Monrós	1956					×	×
	Lychnophaes	Lacordaire	1848		×				
	Dorisina	Monrós	1956		×				
	Lamprosoma	Kirby	1818	×	×				
	Scrophoomorphus	L. Medvedev	1968					×	

All lamprosomatines are highly convex and ventrally flattened [species of Cryptocephalinae, including Chlamisini, sister taxon to lamprosomatines (Farell 1998), are cylindrical and not ventrally flattened], they are shiny and usually iridescent (cryptocephalines are rarely iridescent), their abdominal ventrites are all the same width medially with sternal longitudinal muscles within ventrite 4 [constricted ventrite 4 appearing hidden between ventrites 3 and 5 and sternal longitudinal muscles lacking in cryptocephalines (Kasap and Crowson 1976)]; females lack a fovea or egg-depression on ventrite 5 (present in cryptocephalines); and antennal grooves are present on the prosternum (feature also present in Chlamisini and Ischiopachina of Clytrini).

Upon discovery, Cachiporra could not be placed into any previously recognized lamprosomatine tribe. Clavate antennae are common in many camptosomata (e.g. Chlamisini, Clytrini, Lamprosomatini, but not in Cryptocephalini) (Figs 7, 8), however, Cachiporra is the first known camptosomata with capitate antennae represented by completely fused antennomeres forming an abrupt terminal bulb (capitulum), which is much wider than preceding antennomeres (Figs 16, 18; autapomorphy of Cachiporrini, character 2 state 0). This kind of club is very uncommon in Chrysomelidae; it is known in only a few flea beetle (Alticini) genera commonly living in relatively dense substrate, such as leaf litter or moss cushions (e.g., Clavicornaltica Scherer, Kiskeya Konstantinov and Chamorro-Lacayo) (Konstantinov and Chamorro-Lacayo 2006) and a few Hispini.

**Figures 1–10.**
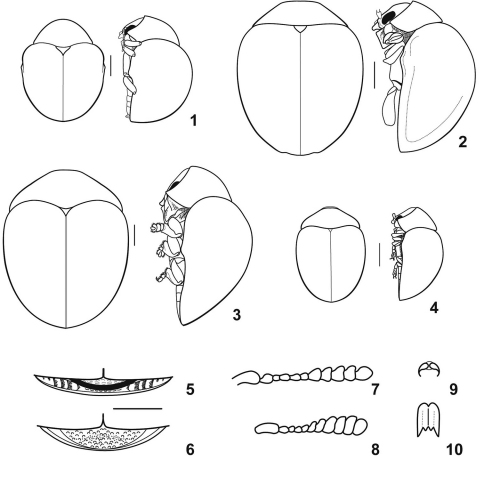
1–4 Lamprosomatinae habiti, dorsal (left) and lateral (right) views, drawn after Monrós, 1956: **1** Sphaerocharis marginicollis (Sphaerocharini) **2** Neochlamys strigicollis (Neochlamysini) **3** Lycnophaes globulosus (Lamprosomatini) **4** Pseudolychnophaes africanus (Neochlamysini); **5–6** Apical margin of ventrite V, drawn after Monrós, 1956: **5** with stridulatory device, Lamprosoma nicaraguensis (Lamprosomatini) **6** without stridulatory device, Sphaerocaris marginicollis (Sphaerocharini); **7–8** Antennae, drawn after Monrós, 1956: **7** Lychnophaes purpureus (Lamprosomatini) **8** Sphaerocharis marginicollis (Sphaerocharini); **9–10** Claws, drawn after Monrós, 1956: **9** Oomorphus floridanus (Lamprosomatini) **10** Sphaerocharis marginicollis (Sphaerocharini).

A phylogenetic analysis of Lamprosomatinae was undertaken to find taxonomic position for Chachiporra within the subfamily. Designation of a new tribe was considered appropriate and justified to accommodate this new genus from Brazil. Current higher-level classification is based on Monrós’s (1956, 1958) interpretation of relationships. The first evolutionary relationship among Lamprosomatinae is here inferred based on cladistic principles.

## Material and Methods

### Taxon sampling and outgroup

This study includes 12 of 14 lamprosomatine genera in 4 tribes (Tables 1 and 3). Exema Lacordaire and Melittochlamys Monrós (Chlamisini) were selected as outgroup to determine character polarity. All examined material is from the National Museum of Natural History (NMNH), Smithsonian Institution, Washington, DC.

### Characters

General morphological terminology follows Konstantinov (1998). Terminology for female genitalia combines Moseyko (2008) and Chamorro-Lacayo et al. (2006), for kotpresse we follow Schöller (2008), and for thorax Chamorro-Lacayo and Konstantinov (2004). Hind wing nomenclature follows Kukalová-Peck and Lawrence (1993). A number of characters used in this analysis have traditionally been used to distinguish among genera and tribes (characters 1, 7, 8, 10, 16, 21 and 22), particularly by (Monrós (1956, 1958). However, the majority of the 26 characters and 70 states are new. All characters have equal weights (Wilkinson 1992) and are unordered (Fitch 1971). Characters were coded based on variations in external morphology among the taxa. Approaches to coding and treatment of characters and missing data is outlined in Chamorro and Holzenthal (2010).

**Table 2.** Characters and states used in the cladistic analysis

1	Canthus of eye: 0, acute; 1, absent (Fig. 17); 2, wide and short; 3, wide and long, longer than half of transverse diameter of eye.
2	Antennomeres 7 to 11 (Figs 7, 8, 16): 0, fused into tight capitulum; 1, free.
3	Antennomeres 6 to 8 (Figs 7, 8, 16): 0, strongly transverse, more than twice as wide as long; 1, about as long as wide; 2, weakly transverse.
4	Base of pronotum in dorsal view: 0, entire; 1, extending posteriorly beyond base of elytra (Fig. 15); 2, extending posteriorly, but not beyond base of elytra.
5	Explanation of lateral margin of pronotum: 0, situated laterally (as in vast majority of Lamprosomatinae and other leaf beetles); 1, situated ventrally, essentially covered from above by expansion of upper side of pronotum laterally (Figs 15, 16).
6	Posterolateral corner of pronotum: 0, extending posterad beyond elytral base; 1, not extending posterad beyond elytral base.
7	Posterior margin of last abdominal ventrite: 0, concave; 1, more or less straight; 2, convex; 3, sinusoidal.
8	Shape of scutellum: 0, acutely triangular; 1, triangular (equilateral); 2, rectangular.
9	Size of scutellum: 0, minute; 1, small; 2, large.
10	Elytral punctation: 0, arranged in regular rows or with tendency to form such rows; 1, completely confused.
11	Sides of elytra: 0, extended into wide long lobe concealing most of middle to posterior part of metepisternite (Fig. 11); 1, extended into wide relatively short lobe not concealing much of metepisternite (Fig. 12); 2, extended into narrow long lobe concealing nearly all posterior part of metepisternite (Fig. 13); 3, extended into wide long lobe not concealing entire metepisternite (Fig. 14).
12	Elytra: 0, covered with bumps; 1, smooth (Fig. 15).
13	Elytral suture: 0, smooth (Fig. 15); 1, dentate.
14	Upper side of beetle body with metallic luster: 0, present; 1, absent.
15	Pronotal and elytral puncture setae: 0, present (Fig. 15); 1, absent.
16	Tarsal claws: 0, bifid and fused (Fig. 10); 1, simple and free (Fig. 9); 2, appendiculate, free, narrowly separated; 3, appendiculate, widely separated.
17	Wing (Fig. 19), number of cells between Cu and A: 0, one; 1, two.
18	Wing, RA 3+4: 0, present; 1, absent.
19	Wing, CuA 3+4 and spur of RP: 0, situated close to each other; 1, placed far away from each other.
20	Epipleura (Figs 11–14) directed: 0, vertically, forming nearly straight line with side of elytron, visible from outside; 1, horizontally, forming nearly straight angle with side of elytron, not visible from outside; 2, vertically, folded behind lateral side of elytron, not visible from outside.
21	Stridulatory file on distal border of last abdominal ventrite: 0, present (Fig. 5); 1, absent (Fig. 6).
22	Pygidium: 0, completely covered by elytra; 1, partly covered by elytra; 2, completely exposed.
23	Sclerotized thin rim of kotpresse: 0, present; 1, absent (Fig. 22).
24	Sclerotized part of spermathecal duct: 0, long, straight, about as wide, but longer than duct of gland (Fig. 20); 1, short, about as long as duct of gland; 2, very long, forming coils, longer than duct of gland; 3, long, consists of narrow and wider parts attached under angle to each other; 4, long, straight, much narrower and longer than duct of gland.
25	Stylus: 0, wider or nearly as wide as long; 1, longer than wide (Fig. 21); 2: absent.
26	Membranous window on apex of coxite (base of stylus): 0, present; 1, absent.

**Table 3.** Matrix of 26 characters for 11 taxa of the Lamprosomatinae and two outgroup taxa

Species name	Character states
	0000000001 1111111112 222222
		1234567890 1234567890 123456
Cachiporra extremaglobosa sp. n.		1001101111 0101010110 121011
Pseudolychnophaes africanus Jacoby	11?0010121 110111??11 12????
Neochlamys strigicollis Jacoby	11?0010121 1101111011 120100
Sphaerocharis marginicollis (Guerin-Meneville)	0100010121 2100101011 11????
Xenoomorphus gingindhlovuanus Monrós	2110011000 2100111011 000120
Oomorphus concolor (Sturm)	2110011000 210111??11 000201
Asisia vietnamica (Medvedev)	2110011010 2101111011 00????
Oomorphoides yoasanicum (Chen)	2110001010 2101121011 000200
Lychnophaes globosus (Olivier)	2110002010 2100111011 000200
Dorisina pusilla (Jacoby)	2111002000 2100111011 000300
Lamprosoma chorisiae Monrós	2111002010 2100121011 000100
Melittochlamys semen (Lacordaire)	3121013221 3001130002 120420
Exema conspersa (Mannerheim)	3122010221 3011130002 120420

### Phylogenetic analysis

The data matrix was created using MacClade 4.08 (Maddison and Maddison 2000) and analyzed using PAUP* (beta test version) (Swofford 2003). Heuristic search was implemented using stepwise addition and 1000 random addition sequence replicates, 5 trees held at each step and Tree-Bisection-Reconnection (TBR) branch swapping algorithm. Nodal support for the preferred topology based on the given dataset was determined by bootstrapping (Felsenstein 1985) and Bremer support indices (Bremer 1988, 1994). Bootstrapping was carried out with 500 replicates and random-taxon addition. Decay or Bremer support indices were calculated by executing in PAUP*a MacClade generated batch file of 20 replicate heuristic searches and random-taxon addition for each constraint tree.

## Results

Five equally parsimonious trees resulted from this analysis (Figs 23–27) of length 55 (CI: 0.80; RI: 0.82; RC: 0.65). A strict consensus tree is shown on Fig. 28. All topological results support the designation of a new tribe, Cachiporrini. Both Bremer and bootstrap values (respectively, separated by comma) strongly support the monophyly of Lamprosomatinae and Lamprosomatini. The placement of Sphaerocharis and the monophyly of Neochlamysini remains uncorroborated in the strict consensus. Majority-rule consensus tree retains a monophyletic Neochlamysini with Sphaerocharini as the sister taxon to Lamprosomantini (Figs 29–31). A majority-rule consensus cladogram is shown with distribution of unambiguous characters in Fig. 29 and ambiguous characters under slow (Fig. 30) and fast (Fig. 31) optimization.

### Key to Tribes of Lamprosomatinae

**Table:** 

1	With stridulatory file (device) on distal border of last ventrite (Fig. 5); last ventrite not excised in shape of arc. Pygidium completely covered by elytra. Scutellum acutely triangular, small to very small (Fig. 3). Elytral punctation arranged in regular rows or with a tendency to form such rows	Lamprosomatini (Worldwide)
–	Without stridulatory file (device) on distal border of last ventrite (Fig. 6); last ventrite varies in shape. Pygidium not completely covered by elytra. Scutellum triangular, equilateral (Figs 1, 2, 4); size either very small or relatively large. Elytral punctation confused (or with a weak tendency to form rows)	2
2(1)	Eyes with deep canthus. Tarsal claws bifid and fused (Fig. 10). Metallic in color, shiny. Pygidium partly exposed, partly covered by elytra	Sphaerocharini Neotropical (South America)
–	Eyes entire, without deep canthus. Tarsal claws simple and free (Fig. 9). Black, moderately shiny or not shiny. Pygidium completely exposed	3
3(2)	Antennae capitate with last 3 or 4 antennomeres tightly fused to form a sphere (Figs 16, 18). Scutellum small. Pronotum basally sinuate, medially extending osterad beyond elytral base. Pygidium exposed. Each puncture of pronotum and elytra with seta	Cachiporrini Neotropical (South America)
–	Antennae not capitate and last 4 antennomeres not fused to form a sphere, instead dentate (Figs 7, 8). Scutellum relatively large. Pronotum not basally sinuate, medially not extending posterad beyond elytral base. Punctures of pronotum and elytra without setae	Neochlamysini Afrotropical

### Cachiporrini

Chamorro & Konstantinov trib. n.

urn:lsid:zoobank.org:act:74B93547-7A5F-430D-9980-46831A35F370

#### Type genus.

Cachiporra Chamorro & Konstantinov

#### Diagnosis.

Body small, about 1.8 mm in length, moderately shiny, black. Head without midcranial and frontal sutures. Frontoclypeus swollen, notched in middle. Eye entire, not notched. Antenna capitate with last 4 antennomeres tightly fused to form sphere (Figs 16, 18).

Pronotum basally sinuate (Fig. 15), medially extending posterad beyond elytral base. Side swollen and bent ventrally so that lateral border not visible from above (Figs 17, 18). Lateral border of pronotum situated close to posterior margin of prosternum leaving hypomera extremely thin and limiting prosternum to small triangle.

**Figures 11–14.**
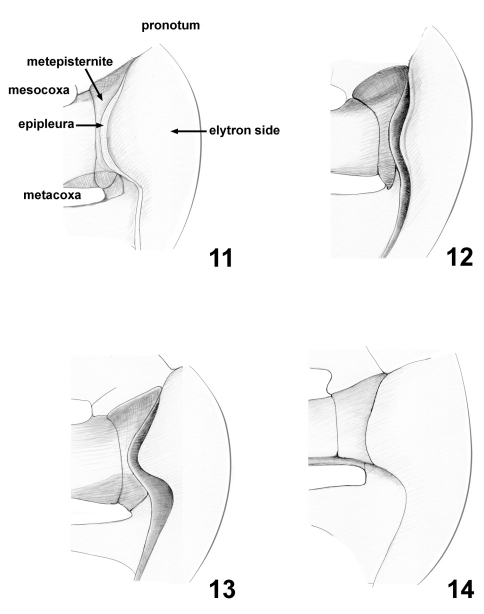
Thoracic characters of Lamprosomatinae and Chlamisini: **11** Cachiporra extremaglobosa **12** Neochlamys strigicollis **13** Sphaerocharis marginicollis **14** Melittochlamys semen

**Figures 15–18.**
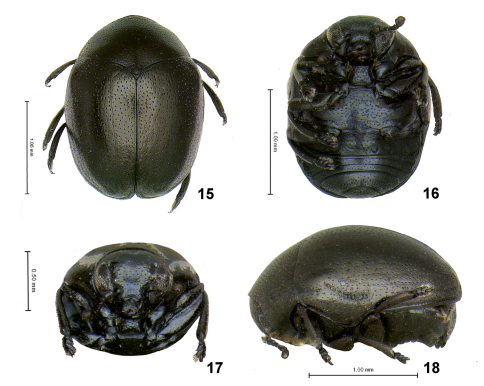
Cachiporra extremaglobosa: **15** dorsal **16** ventral **17** frontal **18** lateral views.

Scutellum triangular, equilateral, small (Fig. 15). Side of elytron with long lobe directed ventrally covering significant part of metepisternite. Epipleura vertical, evenly narrow throughout (Fig. 11). Elytral punctation confused with weak tendency to form rows (Fig. 15). Each puncture of pronotum and elytra with light-colored seta. Tibial apices without long setae and without excavation. Tarsal claws simple and free. Last abdominal ventrite without stridulatory file on distal border. Pygidium exposed (Fig. 18).

### Cachiporra

Chamorro & Konstantinov gen. n.

urn:lsid:zoobank.org:act:FE5E7031-33C2-4340-B822-D6705222A699

Figs 11 15 22 

#### Type species.

Cachiporra extremaglobosa Chamorro & Konstantinov

#### Description.

##### Body small,

1.81–1.85 mm long, 1.43–1.47 mm wide, and 1.01–1.04 mm thick, broadly oval.

##### Color.

Body entirely black with bluish or bronzish tint, antennal capitulum and mouth parts deep reddish brown.

##### Head

flat in lateral view, 1.42 times wider than long in frontal view, completely lacking hairs (except on labrum). Midcranial and frontal sutures absent. Top of frons and bottom of vertex with oval impression. Orbital area swollen and situated above eye level. Internal margin of eye entire, not notched. Distance between eyes 2.64 times as large as transverse diameter of eye. Labrum with three setae on each side placed symmetrically on anterior margin. Antennae extremely capitate with last 4 antennomeres tightly fused to form sphere (Figs 16, 18), inserted slightly above lower eye level with side of antennal socket adjacent to eye margin. Antennomere 1 wide with triangular lobe directed dorsolaterally. Antennomere 2 cylindrical, shorter than wide. Antennomers 3 to 7 dorsoventrally flat, about as long as antennomere 2, but narrower.

##### Prothorax.

Pronotum apically shallowly sinusoidal, basally sinuate, medially extending posterad beyond elytral base. Each pronotal puncture with short seta. Sides swollen and bent ventrally so that lateral border not visible from above. Lateral border situated close to posterior margin of prosternum leaving hypomera extremely thin and limiting prosternum to small triangle. Intercoxal prosternal process in ventral view slightly longer than wide (length to width ratio 1.26), sides constricted above middle, anterior margin evenly concave with obtuse denticle in middle, posterior margin straight. Intercoxal prosternal process in lateral view nearly straight in middle, abruptly bending posterodorsally. Procoxal cavity open posteriorly.

##### Mesothorax.

Scutellum triangular, equilateral, small (Fig. 15). Elytra with well developed humeral calli. Side of elytron with long lobe directed ventrally covering significant part of metepisternite (Fig. 11). Elytral punctation confused, with a tendency to form longitudinal rows. Each elytral puncture bearing short seta. Epipleura narrow. Mesosternum vertical, nearly completely covered with intercoxal prosternal process and anterior process of metasternum separating mesocoxae.

##### Metathorax.

Anterior process of metasternum rectangular with straight anterior margin (Fig. 16). Posterior margin curved. Surface shallowly shagreened with relatively large punctures placed more densely anteriorly.

##### Wing

**Figures 19–22.**
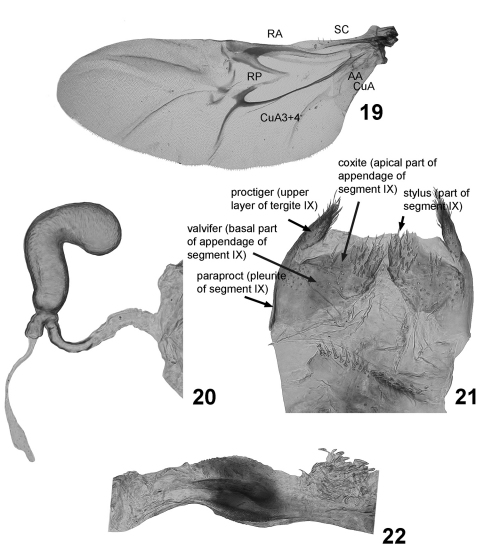
Cachiporra extremaglobosa: **19** wing **20** spermatheca **21** sclerites of female genitalia **22** kotpresse.

fully developed (Fig. 19). Radial (RA) sinusoidal, wide, and strongly sclerotized laterally, bending posteriorly. RP connected to posterior arm of radial cell, divided into two parts: poorly sclerotized median and strongly sclerotized lateral. AA better developed than CuA. RA 3+4 absent. Cu and A form only one complete cell.

##### Abdomen.

First visible abdominal ventrite as long (medially) as rest of abdomen. Second visible ventrite narrowest, half as narrow medially as either third and fourth. Third and fourth of equal length (Fig. 12). Last abdominal ventrite three times longer than preceding, without stridularoty file (device) on distal border. Pygidium exposed.

##### Kotpresse

without dorsal or ventral sclerites with chitinpolster dorsally and ventrally; long lateral fold sclerotized and bent upward and with small denticles (Fig. 22).

##### Legs.

Femora dorsoventrally flat with anterior and posterior sides nearly parallel to each other. Tibiae slightly curved in ventral view gradually widening apically. All tibial apices with small spur, but without long setae and excavation. Tarsal claws relatively small, simple, and free.

##### Female genitalia

(Figs 20, 21). Paraproct (pleurite of segment IX) narrow. Proctiger (upper layer of tergite IX) widely triangular, with setae on their apical margins, moderately sclerotized and narrow membranous stripe between them. Stylus parallel-sided, at base much narrower than long, separated by distinct border from coxite (apical part of appendage of segment IX). Spermatheca without border between pump and receptacle. Base of spermatheca constricted forming short canal branching into gland and duct. Gland and duct relatively short making no loops. Tignum absent.

#### Etymology.

“Cachiporra” is the Spanish word for “billy” club (capitulum). “Cachiporra” alludes to the club-shaped antenna of this lamprosomatine. Feminine.

#### Distribution.

Brazil, Rio Grande do Norte, Natal.

### Cachiporra extremaglobosa

Chamorro & Konstantinov sp. n.

urn:lsid:zoobank.org:act:464C4E20-8CAB-40C4-A072-27D057015464

Figs 11 15 22 

#### Type locality.

Brazil, Rio Grande do Norte, Natal.

#### Description.

Length 1.81-1.85 mm. Color black, with bluish and bronzish tint. Head with frons and vertex shagreened, covered by sparse, sharply defined punctures.

Pronotum strongly shagreened, evenly covered with sharply impressed small punctures, each bearing a single, small seta. Diameter of punctures four to ten times smaller than distance between them.

Elytral surface strongly shagreened, with numerous wrinkles, some of which short and placed diagonally, some exceptionally long and stretched from base of elytron to and beyond middle. Punctures with tendency to form rows.

Female genitalia. Median side of the lateral sclerotization of tergite IX strongly oblique. Stylus attached slightly anteriorly from apex. Receptacle of spermatheca slightly longer than pump, slightly S-shaped with small bump near base and apically. Apex of spermathecal pump bulbous, wider than receptacle and base of pump.

#### Material examined.

Holotype, female: 1) Brazil RG Norte, Papari: III. 1952. M. Alvarengo; 2) F. Monrós Collection, 1959; 3) Holotype Cachiporra extremaglobosa Chamorro & Konstantinov, des. 2010 (NMNH).

Paratype, female: 1) Brazil Natal, R.G. Norte, 24.IX. 1951. M. Alvarengo; 2) F. Monrós Collection, 1959; 3) Paratype Cachiporra extremaglobosa Chamorro & Konstantinov, des. 2010 (NMNH).

#### Etymology.

Named for the extremely globular antennal club (capitulum). The epithet is treated as a noun in apposition.

**Figures 23–28.**
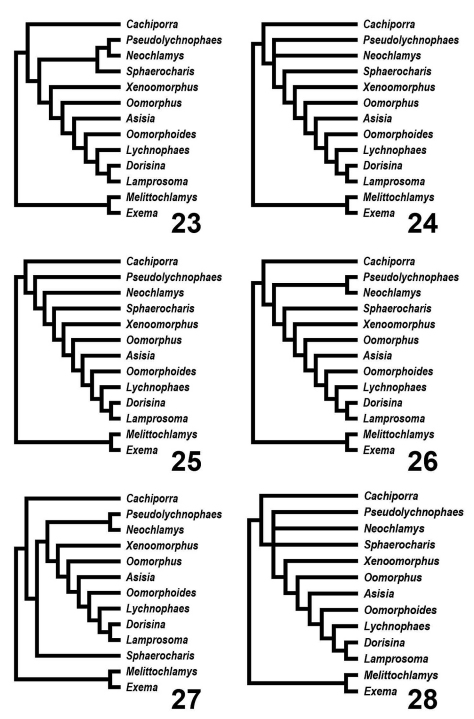
Cladograms of Lamprosomatinae relationships. Outgroup Exema and Melittochlamys (Chlamisini) **23–27** Five equally parsimonious trees **28** Strict consensus of 5 equally parsimonious trees.

**Figure 29.**
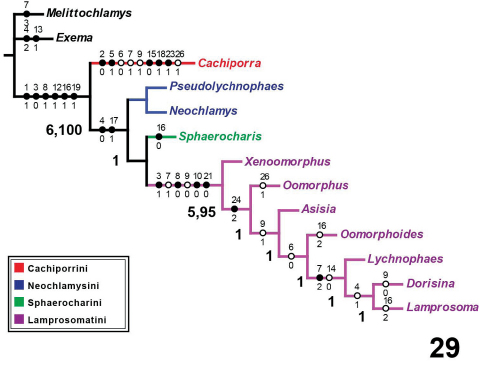
Majority (50%) rule consensus tree with unambiguous character state optimizations. Black circles represent synapomorphies; open circles indicate homoplasious character state transformations. Numbers above correspond to list of characters on table 2; numbers below indicate the state for character indicated above. Bremer support values are indicated by the first numbers on some nodes. Second numbers (only for two clades) separated by a comma indicate bootstrap values (showing only those >5%).

**Figures 30–31.**
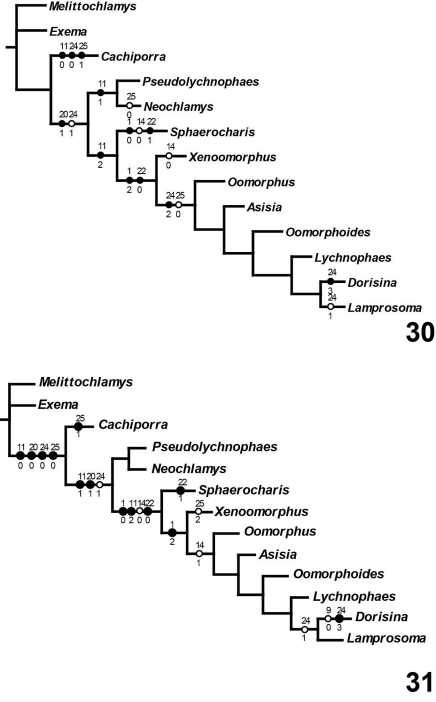
Majority (50%) rule consensus tree. Black circles represent synapomorphies; open circles indicate homoplasious character state transformations. Unambiguous characters deleted **30** Character states under slow (DELTRAN) optimization **31** Character states under fast (ACCTRAN) optimization.

#### Discussion.

Unique features of Cachiporrini include the following: antenomeres 7–11 fused into a tight capitulum (2:0); explanation of lateral margin of pronotum situated ventrally essentially covered from above by expansion of upper side of pronotum laterally (5:1); pronotal and elytral punctures with setae (15:0); wings with RA 3+4 absent (18:1); absence of sclerotized rim of kotpresse (23:1); and stylus of female genitalia longer than wide.

Among various lamprosomatine genera, Cachiporra is superficially most similar in color, size, wing venation, and overall body shape to Oomorphus. The pronotum of Cachiporra is considerably different from that of Oomorphus and all other lamprosomatines in having the sides swollen and bent ventrally so that the lateral border is not visible from above. The lateral border is therefore located very close to the posterior margin of the prosternum, leaving hypomera extremely thin and limiting the intercoxal prosternal process to the shape of a small triangle. In Oomorphus the sides of the pronotum are not bent ventrally and the hypomera and the prosternum itself occupy most of the ventral side of the lateral part of prothorax. Furthermore, dorsally the pronotum in Cachiporra is basally sinuate and medially extending posterad beyond elytral base (Fig. 15) and side of elytron have an extended lobe directed ventrad nearly concealing all of metepisternite (Fig. 11).

Cachiporra has a number of pleisiomorphies also present in African Neochlamysini. These are absence of a stridulatory device (also absent in Sphaerocharini), entire eyes, completely exposed pygidium, and free and simple claws.

Synapomorphies of Lamprosomatinae (Fig. 29) include the loss of canthus of the eye, character 1 state 1 (1:1); antennomeres 6–8 strongly transverse, more than twice as wide as long, 3:0; triangular scutellum, 8:1; smooth elytra, 12:1; tarsal claws simple and free, 16:1; and have CuA 3+4 and spurt RP distant from each other, 19:1. Of the four tribes, Lamprosomatini has the greatest support with at least four synapomorphies and a number of homoplasious character states (Figs 29–31). Sides of elytra extended in wide relatively short lobe, not concealing much of metepisternite (character 11:1) (Fig. 12) supports the monophyly of Neochlamysini under slow optimization (Fig. 30). The transformation of the sides of elytra into a narrow lobe concealing nearly all posterior part of metepisternite (character 11:2) (Fig. 13) is a shared derived feature of Sphaerocharini + Lamprosomatini (Fig. 30) under slow optimization. In Cachiporrini this lobe is wide and long concealing most of middle to posterior part of metepisternite (character 11:0) (Fig. 11); a unique feature of this tribe.

## Supplementary Material

XML Treatment for Cachiporrini

XML Treatment for Cachiporra

XML Treatment for Cachiporra extremaglobosa
